# Neuro-Symbolic Regulatory Retrieval and Compliance Review for Railway Construction Plans Adjacent to Operating Lines

**DOI:** 10.3390/s26196076

**Published:** 2026-09-25

**Authors:** Pengfei Guo, Yalong Xie, Zhihua Wang, Peng Zhi, Lamei Hu

**Affiliations:** Institute of Computing Technologies, China Academy of Railway Sciences Corporation Limited, Beijing 100081, China; pengfeiguo163@163.com (P.G.); zhihuawang1633@163.com (Z.W.); pengzhi16333@163.com (P.Z.); lameihu163@163.com (L.H.)

**Keywords:** railway construction adjacent to operating lines, neuro-symbolic integration, regulatory retrieval, symbolic guardrail, compliance review, human review

## Abstract

Reviewing railway construction plans adjacent to operating lines requires distinguishing clause applicability from documentary evidence of obligation fulfillment. This study integrates neural retrieval and auditing with symbolic applicability and evidence constraints. Structured events drive Best Matching 25 (BM25) and dense retrieval, followed by Cross-Encoder reranking. A pre-applicability guardrail assigns Pass, Reject, or Unknown; for Pass clauses, a neural auditor proposes satisfied, missing, conflicting, or uncertain labels, and a post-evidence guardrail checks their support. Eight anonymized regulatory resources and five projects yielded 809 review units and 1827 event-atomic-obligation instances. With verified event fields, fixed-test Recall@10 and normalized discounted cumulative gain at 10 were 0.928 and 0.878. Independent auditing of 367 reference obligations achieved Macro-F1 0.832 and unsupported determinate-judgment rate 0.019. With automatic inputs, independent-audit Macro-F1 was 0.804. Field evaluation on 277 aligned events yielded measure-set micro-F1 0.899 and evidence-span F1 0.915. Online reference-obligation access was 340/367 (92.64%); classification performance was evaluated separately on the independent audit benchmark. Under frozen regulations and rules, five-fold cross-project Recall@10 and independent-audit Macro-F1 were 0.926±0.008 and 0.827±0.010. These findings support documentary review within the evaluated regime, not verification of on-site execution or approval decisions.

## 1. Introduction

Construction adjacent to operating railway lines is simultaneously subject to constraints arising from construction operations, transport organization, equipment safety, and coordination among multiple parties. Reviewing a construction plan requires not only locating clauses relevant to the current activity across multiple regulations currently in force, but also determining, in light of construction steps, organizational arrangements, and safety measures, whether the plan satisfies requirements regarding responsible parties, actions, objects, conditions, and thresholds. The task, therefore, involves at least two consecutive but fundamentally different decisions: whether a clause applies to the current construction event, and whether the plan evidence is sufficient to support a finding that the corresponding obligation is satisfied, missing, directly contradicted, or still uncertain.

Natural language processing (NLP) and artificial intelligence (AI) have increasingly been applied to engineering-text understanding, risk knowledge extraction, and decision support. Reviews have shown that NLP can support information extraction from engineering standards, analysis of construction documents, and knowledge services for intelligent construction [1]. In railway systems, AI research has addressed operational organization, safety-risk identification, and equipment-condition analysis [2,3]. In related engineering condition-assessment tasks, Guo et al. used post-training and fine-tuning of large language models (LLMs) to integrate fault diagnosis and remaining useful life assessment within a multi-task question-answering framework [4]. For Chinese railway technical texts, Lin et al. performed joint entity and relation extraction from high-speed railway turnout operation-and-maintenance texts, transforming unstructured maintenance records into structured knowledge [5]. Bai et al. constructed a knowledge graph for safety-risk events at railway passenger stations [6]. These studies establish a basis for structured processing of Chinese railway technical texts, but principally address maintenance knowledge, risk-event organization, and knowledge retrieval. Joint determination of construction-plan-to-clause applicability and documentary obligation-fulfillment status remains relatively underexplored.

In automated engineering compliance review, Chinese research has mainly focused on regulatory structuring, building information modeling (BIM) information mapping, and rule-based reasoning. Lin and Guo characterized automated compliance review as a sequence of rule representation, model or data representation, inference execution, and result presentation, and identified complex regulatory conversion and cross-system information matching as persistent engineering challenges [7]. Zhou et al. reviewed BIM-based automated review from the perspectives of regulatory-text information extraction, model semantic enrichment, regulation-to-model matching, and compliance reasoning, noting that more robust text extraction, more general information matching, and more transparent rule code remain necessary [8]. Wang et al. used BIM and ontology technology for rule checking of architectural drawings [9]; Gao et al. performed fire-protection design compliance checking through Industry Foundation Classes (IFC), ontology mapping, and rule-based reasoning [10]; and An et al. used a regulatory-text knowledge graph to support engineering safety management and plan preparation [11]. These studies provide an important foundation for the chain of regulatory structuring, object representation, rule-based reasoning, and result explanation. Their objects, however, are predominantly BIM component attributes, design parameters, or structured models, which do not directly capture the narrative procedures, temporary measures, stage transitions, and cross-party responsibility relationships in railway construction plans.

International research on automated compliance review has further applied ontologies, semantic natural language processing, and logical reasoning to machine-readable representations of regulatory conditions and obligations [12,13,14,15,16]. More recently, Zheng et al. combined LLMs with a nested contract knowledge graph to support knowledge retrieval and risk identification in construction contracts [17]. Vergés et al. integrated LLM-based document parsing, human validation of extracted fields, and deterministic rule execution for regulatory screening of concrete products [18].

At the retrieval layer, Best Matching 25 (BM25) is suitable for matching specialized terms, clause keywords, and identifiers [19]; multilingual dense-representation models such as BGE-M3 can improve recall under semantic reformulation [20]; reciprocal rank fusion (RRF) can combine heterogeneous retrieval results [21]; and retrieval-augmented generation (RAG) constrains the sources of generated responses through external textual evidence [22]. Regulatory-text research nevertheless indicates that textual similarity is not equivalent to legal or regulatory applicability: semantically similar clauses may yield different applicability decisions because of differences in time, responsible party, scope, or exception conditions [23]. Existing regulatory RAG studies have used hybrid retrieval, neural reranking, and multi-passage evidence aggregation for regulatory information retrieval and evidence-grounded responses [24,25,26].

Huang et al. combined structured rail-transit events, hybrid retrieval, Cross-Encoder reranking, and regulatory and case evidence to generate emergency schemes for train-door failures [27]. Building on engineering document review and automated compliance checking [7,8,9,10,11,12,13,14,15,16,17,18], as well as regulatory RAG [22,23,24,25,26], the present contribution is a task-specific integration for narrative construction plans: one decision boundary determines whether a retrieved clause applies; the other determines whether documentary evidence supports a particular obligation label. Separating applicability fields from measures already stated in the plan prevents the latter from defining the retrieval query. The contribution concerns this representation and the two constrained decisions, rather than a new retrieval model or a general-purpose logical reasoner.

In construction-plan review, clause applicability does not imply that documentary fulfillment can be determined. Absence of text supports a missing-content finding only when the examined scope is adequate to establish that absence; otherwise, the status remains uncertain. Absence alone does not establish a direct conflict. Neuro-symbolic research emphasizes the complementarity of neural representations and symbolic constraints in uncertainty management [28], supporting the separation of semantic matching from deterministic rule conditions.

On this basis, this study proposes a neuro-symbolic regulatory retrieval and compliance review method for construction plans adjacent to operating railway lines. Clause applicability determination and obligation-fulfillment determination are explicitly separated, and determinate outputs at the two stages are constrained by a pre-applicability guardrail and a post-evidence guardrail, respectively. The method separates the applicability conditions of a construction event from measures already specified in the plan and their evidence locations; it uses BM25, dense retrieval, and a Cross-Encoder (CE) to produce semantic candidate rankings. A pre-applicability guardrail then processes deterministic conditions concerning validity, discipline, stage, responsible party, spatial scope, and exceptions, and divides candidates into Pass, Reject, and Unknown. Atomic-obligation review is performed only for Pass clauses. A neural auditor produces candidate S/M/V/U labels, whose consistency with clause evidence and plan evidence is checked by the post-evidence guardrail. Unknown applicability, high risk, dependence on external materials, or untraceable evidence triggers human review; retention of applicability-Unknown candidates is subject to the queue-capacity limit.

The main contributions are as follows.

1. A two-level event representation is proposed for construction-plan review. Construction discipline, work activity, construction object, spatial location, construction stage, responsible party, and risk category are organized into an applicability event for clause retrieval and applicability determination. Measures already specified in the plan and their evidence locations are represented independently as audit evidence for downstream obligation-fulfillment determination only. This representation separates, at the data level, whether a clause applies from whether the plan has already fulfilled the clause.
2. A pre-applicability determination mechanism independent of neural relevance scores is constructed. After semantic candidate ranking, candidate clauses are assigned Pass, Reject, or Unknown according to regulatory validity, construction discipline, construction stage, responsible party, spatial scope, and exception conditions. Traceable deterministic applicability conditions directly determine whether a clause enters the automated review evidence set, and applicability-unresolved cases are routed to human review.
3. An evidence-constrained review mechanism for atomic obligations is constructed. The neural auditor produces candidate S/M/V/U labels for applicable clauses. The post-evidence guardrail then checks the responsible party, normative term, action, object, condition, threshold, clause evidence, and plan evidence so that determinate review results satisfy their corresponding evidence conditions. Cases with insufficient evidence, dependence on external materials, or evidence that cannot be traced back retain an uncertain status and enter human review.

## 2. Task Definition and Dataset

### 2.1. Review Task and Label System

In this study, compliance review means assessing whether the submitted plan and its available, explicitly linked materials provide documentary evidence for an applicable obligation. A satisfied label confirms support in those documents, not actual on-site execution, physical safety, or legal approval. Verification of measurements, site conditions, and approval records remains the responsibility of qualified reviewers.

Construction plans adjacent to operating lines generally undergo preparation, technical review, countersignature, revision, and approval. Technical reviewers first identify construction events and risks in a plan, then locate relevant clauses from multiple regulations currently in force, and finally compare the regulatory obligations with construction procedures, organizational arrangements, and safety measures. Because a single plan may cover multiple construction stages and work units, this study defines a construction event as the smallest construction activity described by a relatively stable set of applicability conditions and associated with an independent control requirement. Let the plan document be *D*, from which an event set $E\left(D\right)=\left\{e_{1},e_{2},\ldots ,e_{n}\right\}$ is parsed. Let the regulatory repository be $R=\{r_{1},r_{2},\ldots ,r_{m}\}$, where $r_{j}$ denotes a clause unit that retains its parent heading, anonymized clause identifier, and anonymized source identifier.

For a construction event $e_{i}$, the clause-retrieval task produces a ranked list $\pi_{i}=\left(r_{i1},\ldots ,r_{ik}\right)$, with the objective of placing all manually annotated applicable clauses as high as possible within the top *k* positions. Let $y_{ij}^{\mathrm{ret}}\in \left\{0,1\right\}$ denote the applicability relation between an event and a clause, where 1 indicates that the clause directly constrains or explicitly supports the event and 0 indicates non-applicability. Clause applicability differs from topical relevance. When a clause and an event concern the same construction object but their construction stage, spatial scope, responsible party, regulatory validity, or exception premise is explicitly incompatible, the clause is semantically relevant but inapplicable.

After candidate ranking, the symbolic applicability guardrail outputs $g_{ij}\in \left\{\mathrm{Pass},\mathrm{Reject},\mathrm{Unknown}\right\}$. Pass indicates that no deterministic exclusion condition has been identified and that the clause enters the automated review evidence set. Reject indicates that a clear applicability conflict traceable through a rule identifier, anonymized source identifier, and clause identifier exists; the clause is excluded but an explanatory record is retained. Unknown indicates that event conditions are insufficient, the clause scope is unclear, the cross-reference chain is incomplete, or applicability rules conflict; the clause enters the candidate set for human review. These three states constitute the determination made by the symbolic guardrail. Reject clauses do not enter the review evidence set, and Unknown does not mean inapplicable. A separate review flag $h_{ij}$ indicates whether an event-clause pair requires human review; $h_{ij}$ is independent of applicability state $g_{ij}$.

At the compliance-review stage, atomic obligations are assessed only for event-clause pairs assigned Pass by the pre-applicability guardrail. Evaluation uses a common set of gold-standard Pass instances, including instances for which the system supplies no audit output. Each construction-event-atomic-obligation combination receives $y^{\mathrm{audit}}\in \left\{S,M,V,U\right\}$. *S* (satisfied) denotes documentary support for the required party, action, object, and conditions. *M* (missing) requires an applicable obligation that should be documented, an examined scope sufficient to establish absence, and at least one missing necessary element without a contrary statement. *V* (violation) requires a direct textual conflict with a prohibition, threshold, procedure, or responsible-party requirement. *U* (uncertain) applies when unavailable materials, incomplete context, or relevant truncation prevent a supported judgment. Thus, merely failing to find evidence does not establish *M* or *V*. Applicability Unknown and audit *U* concern different decision stages.

### 2.2. Regulatory Repository, Project Data, and Annotation

The regulatory repository retained eight valid resources, totaling 814 pages, after file-level duplicate detection and version verification of nine original internal files. Owing to data-confidentiality requirements, this study reports only aggregate statistics of regulatory resources and does not disclose identifiable source names, document numbers, organizations, or specific provisions. The eight regulatory resources are denoted uniformly as anonymized source identifiers R1–R8. Model training, experimental evaluation, and result reporting were all conducted with anonymized data.

After layout parsing, construction-related clause filtering, hierarchy restoration, and expert verification, the regulatory repository contained 223 clause units used for retrieval. These 223 clause units were selected and verified from internal regulatory resources according to construction topics, applicability scope, and clause characteristics; they do not represent all clauses in the original files. The filtering criteria were frozen before the project-level training-validation-test split and the determination of gold standards for the test set. Events, applicable clauses, and review labels from the test project were not used during filtering, thereby reducing the risk of test-set-aware data prefiltering.

The fixed-test reference annotation examines every one of the 223 clauses for each of 277 construction events, giving 61,771 distinct event-clause objects. Two experts each completed exhaustive judgments, giving 123,542 judgment records; the separate 367-object gold-standard Pass obligation set received 734 judgments. The event-clause reference universe includes 297 applicable relations and 600 safety-related unresolved relations. The system’s candidate pool does not define the reference universe.

Project data included five mutually independent railway construction projects and 37 project documents. After structural restoration and division into review units, the data contained 809 plan-review units. A plan-review unit is the smallest reviewable content unit in a project document that corresponds to a relatively independent construction activity and control requirement. Pairing construction events with expert-verified regulatory atomic obligations produced 1827 event-atomic-obligation review instances. These quantities are project-level instance statistics and do not represent the number of unique clauses or unique atomic obligations in the regulatory repository.

Project files and regulatory materials were de-identified before processing. Organization names, personal names, contact information, exact mileage, and fields capable of identifying a specific project or document source were excluded from model training and public reporting. The mapping among original documents, de-identified text, and anonymized source identifiers was retained separately by data-management personnel in a restricted environment.

Annotation was performed at two evaluation levels. At the event-clause-pair level, construction-event applicability to regulatory clauses and the Pass, Reject, and Unknown states were annotated. At the event-atomic-obligation level, atomic obligations, plan-measure evidence, and S, M, V, and U labels were annotated. The pre-applicability state was computed once at the event-clause-pair level and inherited by its associated atomic obligations when a clause contained multiple atomic obligations. S/M/V/U labels were defined only for gold-standard Pass event-atomic-obligation instances. Low-confidence event fields were not used as Reject triggers, and non-triggered rules did not imply that a clause was necessarily applicable.

Two annotators with experience in railway engineering, construction safety management, and regulatory review independently completed the annotations. A third expert adjudicated disagreements against the source texts. None of the gold-standard annotators participated in rule implementation. Applicability and S/M/V/U annotation used de-identified regulatory and plan texts without access to system predictions, candidate rankings, or rule_id values. Cohen’s κ was calculated after the two independent annotations and before third-expert adjudication. Evidence-support states used to calculate the unsupported determinate-judgment rate (UER) and applicability-unresolved states used to calculate the Unknown correctness rate (UCR) were independently assigned by annotators who did not participate in rule implementation on the basis of de-identified regulatory and plan texts. System-triggered rule_id values were not used as gold standards during evaluation. Cohen’s κ values for the event-clause applicability relation, the Pass/Reject/Unknown applicability state, and the S/M/V/U labels were 0.88, 0.85, and 0.82, respectively.

Evidence-support assessment examines the candidate judgment and its cited documents rather than treating the guardrail’s acceptance as reference truth. Independent annotation and expert adjudication reduce circular evaluation; they do not eliminate disagreement in regulatory interpretation.

The relationship among construction events, regulatory clauses, and hierarchical labels is shown in Figure 1.

### 2.3. Data Partitioning and Statistics

To prevent templated wording, repeated safety measures, and similar clauses from the same project from entering different data subsets, a project-level fixed split was adopted. Projects 1, 3, and 5 formed the training set, Project 2 the validation set, and Project 4 the test set. All project documents, plan-review units, event-clause pairs, event-atomic-obligation instances, and their evidence from the same project remained in the same subset. The test set was frozen before model architecture, retrieval parameters, and rule thresholds were determined and was used only for final evaluation.

To further assess sensitivity to project differences, project-level five-fold cross-validation was conducted in addition to the main fixed-split experiment. In each fold, one complete project was selected as the external test project, one of the other four projects was selected as the validation project, and the remaining three projects formed the training set. Project documents, plan-review units, event-clause pairs, event-atomic-obligation instances, and their evidence were always partitioned as complete projects, without random within-project splitting. The five rounds followed a cyclic design, so that each project served once as the test project and once as the validation project. This design evaluated the stability of the method when textual formulations, construction content, and applicable-clause distributions varied across projects. Cross-validation was used only for cross-project stability analysis and did not participate in model selection or determination of the main fixed-test results.

The data scale and fixed-split roles are shown in Table 1. Training, validation, and test subsets contained 20, 8, and 9 documents, 471, 168, and 170 plan-review units, and 1069, 378, and 380 event-atomic-obligation instances, respectively. Table 2, Table 3, Table 4 and Table 5 retain results with manually verified event fields, with retrieval and independent obligation auditing reported on their respective evaluation populations. Section 4.6 adds automatic-field results on 277 aligned events and distinguishes the independent 367-instance audit benchmark from the complete online path. Neither input condition evaluates segmentation of review units from raw documents.

The pre-applicability guardrail first assigns Pass, Reject, or Unknown at the event-clause-pair level. When a clause contains multiple atomic obligations, the state is inherited by each obligation. Among the 1827 event-atomic-obligation instances, 22 inherited Reject, 27 inherited applicability-Unknown, and 1778 inherited Pass. Only Pass instances were further labeled S/M/V/U, with 1455, 216, 28, and 79 instances, respectively. Table 1 reports project partitioning and obligation-instance counts; event-clause reference counts are reported separately below.

The fixed test contains $N_{E}=277$ construction events and $N_{G}=297$ gold-standard applicable event-clause relations. Sixty-three units contain one event and 107 contain two, totaling 170 review units. The 297 applicable relations expand to 367 gold-standard Pass obligations; event, relation, and obligation counts are not interchangeable. There are 241 mandatory relations, of which the baseline retrieves 222, yielding mandatory recall 222/241 = 0.921.

The 600 safety-unresolved reference pairs are event-clause objects, distinct from the states of the 13 non-Pass obligations among 380 test obligations. Table S1 distinguishes coverage of all 600 pairs from coverage of the 580 entering CE Top-40.

## 3. Neuro-Symbolic Regulatory Retrieval and Compliance Review Method

### 3.1. Overall Framework

Figure 2 separates offline repository and rule preparation from online retrieval and review. Offline processing segments clauses, encodes applicability conditions and atomic obligations, verifies them with experts, and freezes their versions and indexes. Online processing constructs an applicability event, ranks clause candidates, applies the pre-applicability guardrail, and audits the atomic obligations of Pass clauses. The post-evidence guardrail then checks candidate labels. Routing conditions are specified in Section 3.5; the output retains the event, clause, applicability state, evidence, candidate and final labels, review flag, and recommendation.

Neural models address semantic ambiguity, whereas symbolic rules enforce documented deterministic constraints. Aggregate experiments evaluate retrieval, applicability filtering, and audit outputs. Table S2 reports the error distribution, seed-specific V counts, and two failure cases.

### 3.2. Structured Representations of Events and Clauses

To separate clause applicability from plan-fulfillment evidence, construction events are represented at two levels. The applicability event is defined as

(1) $$e^{\mathrm{app}}=\langle P,A,O,L,T,R,K\rangle$$

In Equation (1), *P*, *A*, *O*, *L*, *T*, *R*, and *K* denote construction discipline, work activity, construction object, spatial location, construction stage, responsible party, and risk category. This representation supports retrieval and applicability determination without measures already specified in the plan.

Audit evidence is represented as

(2) $$e^{\mathrm{audit}}=\langle e^{\mathrm{app}},M,E_{M}\rangle$$

In Equation (2), M is the set of measures stated in the plan and $E_{M}$ contains their source spans and locations in sections, tables, or lists. Both are used only for downstream review and are excluded from applicable-clause retrieval.

Each regulatory clause is represented as

(3) $$c\left(r\right)=\langle C_{r},O_{r}^{\mathrm{obl}},H_{r}\rangle$$

In Equation (3), $C_{r}$ records applicability conditions; $O_{r}^{\mathrm{obl}}$ contains the atomic obligations; and $H_{r}$ records the anonymized source and clause identifiers, validity, version, hierarchy, and cross-references.

Event parsing uses the Qwen3-8B LLM [29], structured prompts, and controlled vocabularies. Its input contains the current paragraph, parent heading, one neighboring paragraph on each side, and current table header, capped at 1024 tokens. Non-thinking mode, disabled sampling, temperature 0, and a 512-token output cap produce the seven fields, measures, and source locations in JavaScript Object Notation (JSON) Schema. A single deterministic parse is used; self-reported model probabilities are not confidence scores. Schema failures, unmapped controlled terms, and untraceable spans flag low-confidence fields. Manual correction is limited to the verified-input condition; automatic inputs retain parsing failures without manual field correction. The complete online path does not inject gold-standard clauses. The independent audit in Section 4.6 instead supplies fixed gold-standard targets under the protocol in Section 4.1.

Regulatory clauses are uniformly represented by applicability conditions, atomic obligations, and hierarchical metadata. Applicability conditions record discipline, action, object, location, stage, responsible party, and risk scope. Atomic obligations record the responsible party, normative term, action, object, condition, threshold, and exception. Hierarchical metadata record the anonymized source identifier, clause identifier, validity, version identifier, clause hierarchy, and cross-reference relationships. Compound clauses are retained as retrieval-display units but are split into atomic obligations at the review stage.

### 3.3. Candidate-Clause Retrieval and Semantic Ranking

To balance specialized-term matching and semantic reformulation, BM25 [19] and dense retrieval are used in parallel. A query consists of the event text and its *P*, *A*, *O*, *L*, *T*, *R*, and *K* fields and does not include measures already specified in the plan. Candidate clauses consist of their text, parent headings, and anonymized source identifiers. BM25 uses jieba 0.42.1 Chinese word segmentation together with a domain dictionary covering construction disciplines, work activities, railway equipment, responsible parties, construction grades, risk categories, synonyms, and abbreviations, with $k_{1}=1.2$ and b=0.75. Dense retrieval uses the frozen BAAI/bge-m3 bi-encoder [20], with a 1024-dimensional output and a maximum input length of 256 tokens. Query and clause vectors are L2-normalized and ranked by cosine similarity, and a Facebook AI Similarity Search (FAISS) IndexFlatIP index is used (https://faiss.ai/, accessed on 20 September 2026).

Reciprocal rank fusion (RRF) is used only to produce a fused candidate set. The Cross-Encoder performs the final ranking of this set, and its scores are not subsequently reweighted with RRF scores.

The top 40 candidates from RRF enter Cross-Encoder reranking. The reranker uses BAAI/bge-reranker-v2-m3 to perform fine-grained semantic matching between event text with applicability conditions and clause title, body, and anonymized source identifier. Each positive example is paired with one random negative, one top-ranked BM25 negative, and one top-ranked dense-retrieval negative. The three negative types have a 1:1:1 ratio, yielding an overall positive-to-negative ratio of 1:3. Applicability-Unknown instances are not treated as definite negatives.

For a candidate clause $r_{j}$, its fused score is jointly determined by its ranks in the BM25 and dense retrieval results:

(4) $$s_{\mathrm{rrf}}\left(r_{j}\right)=\sum_{g\in \{\mathrm{bm}25,\mathrm{dense}\}}\frac{w_{g}}{k_{0}+{\mathrm{rank}}_{g}\left(r_{j}\right)}$$

In Equation (4), ${\mathrm{rank}}_{g}\left(r_{j}\right)$ is the clause rank in channel *g*, $w_{g}=1$ for both channels, and $k_{0}=60$. Each channel returns 50 candidates; their union is deduplicated and fused, and its top 40 enter Cross-Encoder reranking. Reranking changes the order, not membership, of this 40-clause pool.

The Cross-Encoder produces semantic candidate rankings for event-clause pairs. Regulatory validity, construction stage, responsible party, spatial scope, and exception relations are retained as independent structured conditions and are used for applicability determination in the subsequent pre-applicability guardrail.

The Cross-Encoder is lightly fine-tuned with binary cross-entropy. The training batch size is 4, gradient accumulation is 4 steps, and the effective batch size is 16. AdamW is used with a learning rate of $2\times 10^{-5}$, weight decay of 0.01, a warm-up ratio of 0.10, and a maximum gradient norm of 1.0, together with 16-bit floating-point (FP16) mixed precision. Training runs for at most 3 epochs with early stopping patience of 1 epoch; the checkpoint is selected according to validation normalized discounted cumulative gain (nDCG) at 10. Random seeds are 42, 2024, and 2026.

### 3.4. Pre-Applicability Guardrail for Clause Applicability Determination

The pre-applicability guardrail takes event fields, clause applicability conditions, and hierarchical metadata as input. Reject requires a traceable validity or scope conflict after the relevant validity, exception, priority, and reference conditions have been resolved. When an event satisfies a valid exception, the exception rule takes precedence over the general rule; an unresolved exception premise does not justify immediate rejection under the general rule. It outputs Unknown when necessary fields are missing, cross-reference chains are incomplete, version relationships are unclear, or conflicting rules cannot be resolved. Every determination retains a rule_id, anonymized source identifier, clause identifier, triggering condition, and explanation.

Symbolic rules are derived from applicability conditions, atomic obligations, and hierarchical metadata in the frozen regulatory repository. The conversion process comprises structured-candidate generation, mapping of normative terms to the controlled vocabulary, rule-template instantiation, item-by-item expert verification, and version freezing. Exceptions, cross-clause cross-references, and version relationships that cannot be reliably represented using templates are supplemented by experts. To avoid disclosure of internal regulatory structures, the distribution of rules across anonymized source files and actual rule texts are not reported.

Each rule records its rule_id, anonymized source identifier, clause identifier, version and validity state, applicability-condition predicates, rule action, priority, exception conditions, cross-reference dependencies, and explanation template. Rule actions include Pass, Reject, Unknown, and downstream label constraints. The symbolic guardrail and neural relevance ranking operate in independent decision spaces. A conflict concerning validity, discipline, stage, responsible party, or spatial scope produces Reject only if it remains applicable after the relevant exceptions and priority relations are resolved. Insufficient necessary conditions or unresolved rule relationships produce Unknown. Candidates with resolved conditions and no applicable exclusion enter Pass; mere absence of a rule trigger does not resolve missing prerequisites. Deterministic applicability rules do not participate in neural-relevance-score weighting.

The documented priority order is validity state, explicit exception, regulatory hierarchy, special-versus-general provision, and old-versus-new version relationship. A general-rule incompatibility cannot be finalized as Reject before a potentially applicable valid exception and its references are resolved. A traceable conflict under the resolved scope produces Reject; missing prerequisites or an unresolved priority conflict produce Unknown with the competing rule identifiers retained.

The pre-applicability guardrail is executed on the top 40 Cross-Encoder candidates. After Reject candidates are removed, the system preserves the original ranking and advances through subsequent candidates until Pass Top-10 is formed or the candidate list is exhausted. Unknown candidates retain their original ranking and enter an independent human-review queue. Reject candidates retain rule explanations but do not enter the automated review evidence set. This rank-preserving backfill mechanism allows applicable clauses initially ranked below the top 10 to enter the final evidence set, distinguishing the proposed method from post-hoc filtering applied only to the Cross-Encoder Top-10.

The main Unknown queue retains the five highest-ranked Unknown candidates per event. Table S1 evaluates this as a capacity limit: safety-reference pairs outside the retained queue count as omissions. Increasing queue capacity does not change the Pass evidence set. The independent review flag is defined in Section 3.5.

The determination and routing process of the pre-applicability guardrail is shown in Figure 3.

### 3.5. Evidence-Constrained Atomic-Obligation Review and Human Review

The neural auditor uses Qwen3-8B, loaded locally with 4-bit NormalFloat (NF4) quantization and without supervised fine-tuning. It runs in non-thinking mode with stochastic sampling disabled, a temperature of 0, and maximum input and output lengths of 4096 and 512 tokens, respectively. During online review, the neural auditor processes only clauses assigned Pass by the pre-applicability guardrail; Unknown and Reject clauses do not enter determinate automated review. The common evaluation set for the four-class experiment consists of gold-standard Pass instances, as specified in Section 4.1.

At this stage, the event, Pass clause, atomic obligation, and plan evidence together form the traceable evidence context provided to the neural auditor. The auditor is restricted to this context, and its output is a review recommendation rather than an automated approval decision.

The plan-evidence scope is fixed before the experiment as the paragraph containing the current plan-review unit, the parent heading, two adjacent paragraphs before and after that unit, and the tables and attachment fragments directly cited by the unit. Table input includes headers and the rows corresponding to the current review unit. Attachments include only relevant fragments explicitly cited in the text. Evidence text is limited to 2048 tokens. When the input exceeds the length limit, the current atomic obligation, clause text, current paragraph, and directly cited evidence are retained first, followed by adjacent paragraphs in order of distance. Truncated content cannot be used as evidence for a determinate label.

Truncation is relevant to the label when it removes material needed to establish fulfillment, absence, or a direct conflict. Such uncertainty requires *U* and human review; truncation of unrelated context alone does not force *U*. A missing-content judgment must identify the examined scope and missing element rather than invent a positive evidence span for an absent measure. The truncation analysis below distinguishes truncation frequency from its effect on M/U predictions.

The neural auditor returns a candidate S/M/V/U state under the label definitions in Section 2.1. Acceptance as a determinate output requires the corresponding clause evidence, plan evidence or documented examination scope, and obligation-element conditions. Unverifiable candidates remain *U* or enter human review. The task instruction restricts the model to the supplied event, Pass clause, current obligation, and plan evidence; it prohibits external knowledge, unsupported thresholds, and inferring *V* from absence alone.

The model returns a fixed JSON structure containing label, clause_evidence, plan_evidence, and missing_elements. It also contains conflict_elements, reason, suggestion, and review_required. Evidence fields must contain an anonymized source identifier or plan-location identifier; real regulatory names, document numbers, and organization names are not exposed in the text or system output.

Model outputs are first checked for Schema conformance, controlled labels, evidence traceability, and field length. When initial parsing fails, only one deterministic format repair is performed; the repair prompt may not add new evidence or change the original basis for the judgment. If parsing fails again, a required field is missing, or evidence cannot be traced back, the candidate label is changed to *U* and review_required=true is set.

The post-evidence guardrail checks source traceability, obligation-element coverage or conflict, label-specific support, and preservation of the original normative term, unit, responsible party, and version in the recommendation. For *S* and *V*, support requires locatable fulfillment or direct-conflict evidence. For *M*, support requires the applicable obligation, an adequate examined scope, and the identified missing element; an absent measure need not have a positive supporting span. Unavailable material that prevents this check yields *U*. Recommendations retain source and clause identifiers, applicability conditions, normative terms, plan locations, and responsibilities; unsupported expressions are removed or left for human verification.

The human-review flag h=1 is triggered by applicability Unknown, high-risk Pass, unavailable required materials, unresolved rule conflicts, format-repair failure, untraceable evidence, or a candidate label disallowed by the evidence rules. This flag is independent of applicability and audit labels, so a supported *S* can still require review. Technical reviewers accept, modify, or reject recommendations and record reasons; their decisions do not enter automated classification scores. Multiple triggers are retained in the task explanation when combined into one review flag. Section 4.7 reports obligation counts, task counts, and review-unit counts separately; the routing fraction does not measure time saved.

High-risk status is determined by the structured risk field K and high-risk markers in the frozen rule base, rather than an LLM judgment. Experts defined the routing policy from the regulations before testing and froze it for evaluation; it was neither adjusted on test outcomes nor selected through validation-set search to increase F1.

The neural audit, post-evidence guardrail, and human-review routing are shown in Figure 4.

## 4. Experiments and Results

### 4.1. Comparative Methods, Metrics, and Implementation Settings

Four research questions were investigated:

- **RQ1:** Can semantic candidate retrieval and applicability filtering provide a regulatory evidence set with high coverage and high ranking quality among top-ranked results for downstream review?
- **RQ2:** Can the pre-applicability guardrail effectively reduce hard-conflict clauses in the final evidence set while controlling false rejection of applicable clauses?
- **RQ3:** Can evidence-constrained review improve the quality of S/M/V/U determinations and reduce determinate judgments lacking supporting evidence?
- **RQ4:** Under complete project-level isolation, can the proposed method maintain stable retrieval and review performance across independent construction projects?

Five methods are compared for clause retrieval: BM25, Dense, Hybrid, Cross-Encoder, and Proposed. Hybrid fuses BM25 and Dense with RRF; Cross-Encoder reranks the fused candidates; and Proposed adds the complete pre-applicability guardrail after Cross-Encoder ranking.

The audit comparison retains Direct-LLM and Hybrid-RAG and uses a 2×2 guardrail ablation around the same neural pipeline: Neural-only (neither guardrail), Neural + Applicability Guardrail (pre only), Neural + Evidence Guardrail (post only), and Proposed (both). These configurations share fixed target clauses, target atomic obligations, the base auditor, and the independent-audit evaluation instances. The pre-guardrail determines admission for the current target without substituting another clause. Disabling the post-guardrail removes semantic evidence checks but retains JSON parsing, controlled-label checks, and failure accounting. A standalone symbolic S/M/V/U auditor is not implemented: the symbolic components constrain applicability and evidence admission but do not independently interpret free-text measures for the four-class task. Their contribution is examined through this ablation and the applicability-rule analysis in Table 3.

The independent audit benchmark supplies the gold-standard target clause and target atomic obligation for each of 367 reference Pass instances; it does not require online retrieval to rediscover the target. With the pre-guardrail enabled, only a Pass decision admits that fixed target to neural auditing. An erroneous Reject or Unknown prevents auditing and contributes a no-output false negative to the reference class, without substituting another clause. The automatic-input condition replaces all nine parsed outputs without manual correction: P/A/O/L/T/R/K, the measure set M, and its evidence locations $E_{M}$. The first seven fields serve retrieval and pre-applicability checks; the last two supply audit evidence. Retrieval scores and this gold-target-assisted independent audit are evaluated separately and do not constitute complete online classification.

To ensure fair comparison, all review methods use the same base language model, task definition, output Schema, decoding parameters, and plan-evidence scope. Regulatory-evidence context and guardrail settings follow the method definitions; the fixed-target independent-audit protocol applies to the common evaluation population.

Retrieval reports Recall@10, normalized discounted cumulative gain (nDCG) at 10, mandatory-clause recall, and critical miss rate. Recall pools retrieved gold-standard applicable relations over the common reference count; the fixed test contains 297 such relations. Mandatory recall uses 241 expert-identified mandatory relations. Critical miss is the proportion of 277 test events missing at least one critical reference clause from Top-10. The hard-conflict rate (HCR) is event-level, whereas false rejection rate (FRR) and Unknown correctness rate (UCR) concern event-clause pairs. Reference-Unknown pairs are assessed separately through UCR and safety-reference coverage; they are not confirmed applicable hits. Four-class scores use gold-standard Pass event-atomic-obligation instances, with Macro-F1 defined in Equation (5). Routing is reported separately from classification.

Metrics are computed from unrounded run-level results and rounded only after aggregation. All methods within each comparison use the same reference populations and metric definitions. Frozen retrieval outputs and deterministic rule configurations retain identical counts across runs.

(5) $$\text{Macro-F}1=\frac{1}{4}\sum_{c\in \{S,M,V,U\}}{F1}_{c}$$

Here, ${F1}_{c}$ is the harmonic mean of precision and recall for class *c*; each of the four reference classes receives equal weight.

In Equation (6), E contains all test events and $E_{\mathrm{hc}}$ contains those with at least one independently identified hard-conflict clause in Top-10. Each event contributes at most once to HCR. $G_{\mathrm{in}}$ contains gold-standard applicable event-clause pairs entering the guardrail, and $G_{\mathrm{rej}}$ contains those incorrectly rejected. Obligation expansion does not multiply these pairs:

(6) $$\begin{array}{cccc} \mathrm{HCR}@10 & =\frac{\left|E_{\mathrm{hc}}\right|}{\left|E\right|}, & \mathrm{FRR} & =\frac{\left|G_{\mathrm{rej}}\right|}{\left|G_{\mathrm{in}}\right|} \end{array}$$

UCR is the proportion of system-Unknown pairs independently confirmed as applicability-unresolved, not coverage of all unresolved pairs. The baseline denominator precedes the Top-5 cap: 557 of 639 system-Unknown pairs are reference-unresolved, giving UCR = 0.872. The unsupported determinate-judgment rate (UER), Equation (7), uses all emitted S/M/V judgments as D and their independently unsupported subset as $D_{\mathrm{unsupported}}$. In the independent 367-instance benchmark, each run has 342 determinate outputs and the three unsupported counts are 6/6/7, giving mean UER = 19/1026 = 0.019. UER for the 3421 online outputs requires a separate count. Audit *U*, missing outputs, and human-review flags alone are not determinate judgments. Neither rule identifiers nor post-guardrail acceptance define the reference support state:

(7) $$\mathrm{UER}=\frac{\left|D_{\mathrm{unsupported}}\right|}{\left|D\right|}$$

The false violation rate (FVR), Equation (8), uses reference M/U instances as $G_{MU}$ and their subset predicted as *V* as $G_{MU\to V}$. Its fixed-test denominator is 44+17=61 per run, not all 367 instances or all predicted violations. It does not count a reference *S* incorrectly predicted as *V*; those errors remain in classification scores and the full *V* false-positive count:

(8) $$\mathrm{FVR}=\frac{\left|G_{MU\to V}\right|}{\left|G_{MU}\right|}$$

All trainable models were run independently with random seeds 42, 2024, and 2026. Table 2, Table 3 and Table 4 report mean results across the three runs in the main fixed-split experiment. Because the main fixed-split experiment contains only one independent test project, no cross-project significance test is performed on that fixed split, and the three random seeds are not treated as independent project repetitions. For each project-level test fold, the same three seeds were run independently, and the mean of the three runs was first calculated for that test project. The cross-project mean and standard deviation were then calculated from the five independent test-project means. Random-seed repetitions were not treated as independent project samples in cross-project statistics.

In project-level five-fold cross-validation, the Cross-Encoder was fine-tuned in each fold only with event-clause positive samples from the three training projects and with the random negatives, top-ranked BM25 negatives, and top-ranked dense-retrieval negatives constructed as described in Section 3.3. The validation project was used only for early stopping and checkpoint selection. The test project did not participate in positive-sample construction, negative-sample construction, checkpoint selection, or threshold adjustment. BM25, Dense, and Qwen3-8B used the same frozen models and parameters as in the main fixed-split experiment. The symbolic rule base, controlled vocabulary, retrieval candidate sizes, and guardrail execution logic were uniformly frozen before the five-fold experiment and were not modified according to any test project during testing.

The reported experiments used Ubuntu 22.04, an Intel Core i9-13900KF central processing unit (CPU; Intel Corporation, Santa Clara, CA, USA), an NVIDIA GeForce RTX 3080 Ti graphics processing unit (GPU; NVIDIA Corporation, Santa Clara, CA, USA), 64 GB memory, and Python 3.9. The Cross-Encoder input limit was 512 tokens; retrieval and training settings are specified in Section 3.3, and parsing and audit settings in Section 3.2 and Section 3.5. Manuscript translation, bilingual revision, and schematic preparation were assisted by OpenAI Codex under the supplied revision plan; this assistance did not provide experimental measurements.

The main four-class experiment used only real project samples. Synthetic violation samples were not included in the training set, validation set, or Macro-F1 calculation of the main experiment. Any synthetic-violation stress test should be fully separated from the main experiment and reported separately.

### 4.2. Clause Retrieval Results

Clause-retrieval results are shown in Table 2. The proposed method achieved Recall@10 of 0.928 and nDCG@10 of 0.878, improving on Cross-Encoder by 0.030 and 0.038, respectively. The critical miss rate decreased from 0.087 to 0.052.

Rank-preserving backfill admits applicable clauses from CE ranks 11–40 after exclusions among earlier candidates. Across three runs, the original CE Top-10 contains 800 gold-standard hits; three are lost and 30 are backfilled, producing 827 final hits. The effective backfill share is B/H=30/827=3.63%, where *B* counts final hits originally ranked 11–40 and *H* counts all final hits. This pooled ratio differs from the net Recall@10 increase of 0.030. Of the 30 backfilled hits, 26 originate at ranks 11–20, four at 21–30, and none at 31–40.

Each run contains 285 applicable relations in CE Top-40 out of 297 references, giving Recall@40 = 0.960; three false rejections give FRR = 3/285 = 0.011. The unchanged RRF pool has the same Recall@40 because reranking changes order, not membership. CE Top-10 hits are 267/267/266, and final Top-10 hits are 276/276/275. Thus, pool recall exceeds final recall under the common reference denominator.

### 4.3. Effect of the Pre-Applicability Guardrail

The complete pre-applicability guardrail reduced HCR@10 from 0.312 to 0.041 and increased Recall@10 from 0.898 to 0.928. FRR and UCR were 0.011 and 0.872, respectively. These results show that the pre-applicability guardrail reduced hard-conflict-clause retention while maintaining a low false-rejection rate for applicable clauses. Applicability-Unknown retention remains subject to the queue-capacity limit. The results support RQ2.

### 4.4. Four-Class Review and Post-Evidence Guardrail Results

The independent four-class benchmark uses 367 gold-standard Pass event-atomic-obligation instances: 300 *S*, 44 *M*, 6 *V*, and 17 *U*. An instance with no S/M/V/U output contributes a false negative for its reference class; it is neither omitted nor credited as a correct *U*. Human-review decisions do not replace automated outputs. These independent-audit results are separate from the online run, whose reference access and computational workload are reported in Section 4.7.

Table 4 reports independent four-class review and post-evidence guardrail results. Proposed achieved Macro-F1 0.832, with S/M/V/U-F1 of 0.978/0.854/0.727/0.769, UER 0.019, and FVR 0.016. Pre-only achieved Macro-F1 0.771, post-only 0.800, and Neural-only 0.739.

Relative to Neural-only, Proposed improves Macro-F1 by 0.093 and reduces UER and FVR by 0.046 and 0.017. Relative to post-only, its *M*-F1 and *U*-F1 increase by 0.054 and 0.069, *S*-F1 by 0.007, and Macro-F1 by 0.032; *V*-F1 remains 0.727 and UER falls by 0.009. These are descriptive differences on the independent benchmark. Both configurations have V true-positive/false-positive/false-negative counts of 4/1/2 at seeds 42/2024/2026. Both predict V01–V04 as V, V05 as S, and V06 as U. This agreement concerns the six reference V cases and does not establish identical false-positive instances. The V-F1 gain over Neural-only comes from fewer false positives, not more detected V cases. The four configurations evaluate the guardrails separately and jointly; these descriptive differences do not establish a statistically significant interaction. Because this benchmark fixes target clauses, the classification differences do not measure retrieval-backfill gains; without paired outputs on common instances, they cannot be attributed entirely to pre-guardrail admission.

### 4.5. Cross-Project Stability Under a Fixed Regulatory Regime

Project-level five-fold cross-validation assesses stability across projects sharing the same frozen 223-clause repository, symbolic rule base, and controlled vocabulary. Each fold holds out one test project, uses the next project cyclically for validation, and trains the Cross-Encoder on the remaining three. The fold result is the mean over seeds 42, 2024, and 2026. Project 4, therefore, uses Project 5 for validation in this analysis, whereas the fixed main experiment uses Project 2; the two Project 4 results need not coincide. This design does not test transfer to an unseen regulatory regime or a changed rule base.

Table 5 reports the results when each of the five independent projects was used as the test project. The proposed method obtained Recall@10 values from 0.915 to 0.934 and Macro-F1 values from 0.812 to 0.837 across the five projects, with cross-project means of 0.926±0.008 and 0.827±0.010, respectively. HCR@10 and UER ranged from 0.036 to 0.050 and from 0.017 to 0.024, respectively, with cross-project means of 0.042±0.006 and 0.020±0.003. Within the current set of five independent projects, these results indicate a degree of cross-project stability in retrieval coverage, applicability constraints, and evidence-constrained review. However, this conclusion requires further validation on a larger set of independent projects because of the number of projects and the sample size of low-frequency classes.

### 4.6. Event Extraction and Automatic-Input Evaluation

Field evaluation uses the same 170 presegmented Project 4 review units: 63 contain one reference event and 107 contain two, giving 277 reference events. Parser fields, measures, and evidence spans are compared with their references under one-to-one event alignment. The evaluation concerns field quality on aligned events; raw-document segmentation and event discovery are outside this comparison.

Table 6a reports accuracy for scalar P/L/T, set micro-F1 for A/O/R/K and measures, and event-macro character-span overlap F1 for evidence. Each field has 277 event-level positions; multivalued scores pool label items. Missing spans receive zero. Schema conformance is reported separately at the review-unit level.

The five multivalued fields A/O/R/K/measures have 420/350/360/330/700 reference label items and 409/343/348/312/662 predicted items. Evidence spans comprise 235 exact matches, 24 cases with overlap F1 of 2/3, four with 3/5, and 14 missing spans, giving (235+24×2/3+4×3/5)/277=0.915. Exact-span accuracy is 235/277 (84.84%); traceable spans account for 263/277 (94.95%). These quantities use different criteria and are reported separately.

All seven applicability fields are simultaneously correct for 199/277 events (71.84%); all nine fields are correct for 180/277 (64.98%). The remaining 97 events (35.02%) require field correction, comprising 244 erroneous positions out of 2493 (9.79%), or 2.52 corrections per affected event. These counts quantify correction volume, not measured human editing time.

Automatic inputs yield Recall@10 0.903, HCR@10 0.055, independent-audit Macro-F1 0.804, and UER 0.028 (Table 6b). The automatic condition records 46 hard-conflict events across three runs, giving HCR@10 = 46/831 = 0.055. Relative to verified inputs, Recall decreases by 0.025 and Macro-F1 by 0.028, while UER increases by 0.009. Each of the three audit runs retains all 367 reference obligations, with 23/23/24 errors including three no-output failures per run. Unsupported determinate counts are 10/9/10 over 343/343/340 determinate outputs. Review routing is 62/367 (16.89%) for automatic inputs and 52/367 (14.17%) for verified inputs. This independent benchmark is separate from the online workload below.

### 4.7. Targeted Validation and Practical Cost

#### 4.7.1. Candidate and Queue Sensitivity

Table S1 varies one parameter at a time around the main setting: 25/50/100 candidates per channel, fused-pool sizes 20/40/60, and RRF constants 30/60/90. For N=25, seeds 42/2024/2026 yield 271/272/271 final applicable hits out of 297 per run. Pooled Recall@10 is 814/891 = 0.914, HCR@10 is 38/831 = 0.046, and critical miss is 53/831 = 0.064; mean nDCG@10 is 0.864. Increasing *N* from 50 to 100 changes Recall@10 from 0.928 to 0.929. Reducing the fused pool from 40 to 20 lowers Recall@10 to 0.906, whereas increasing it to 60 gives 0.932. Actual pool sizes are 20/40/60. RRF constants 30 and 90 give Recall@10 0.926 and 0.928. These perturbations characterize the main setting without retuning it on the test set.

The complete independent safety-unresolved reference contains 600 pairs in the 61,771-pair universe: 20 lie outside CE Top-40, 17 are misassigned Pass, six are misassigned Reject, and 557 are correctly assigned Unknown. Within-pool coverage uses 580 references, and whole-reference coverage uses 600. Capacities 5/10/all retain 512/540/557 references, yielding within-pool coverage 88.28/93.10/96.03% and whole-reference coverage 85.33/90.00/92.83%. Capacity alone omits 45/17/0 correctly identified pairs; 43 upstream omissions persist without a cap. Pre-cap UCR is 557/639 = 0.872. The Top-5 cap omits 45 safety-unresolved pairs already identified as Unknown; the reported queue does not guarantee complete retention of such cases. These results characterize queue retention, not subsequent recovery of omitted cases or completed human review.

#### 4.7.2. Evidence Window and Errors

Table 7 compares nominal plan-evidence caps of 2048 and 3072 tokens on the independent 367-obligation benchmark. The evidence allowance is constrained by min{W,max(0,4096−H)}, where *H* is all non-plan-evidence input and *W* is the nominal evidence cap. The comparison retains the same clauses, obligations, evidence-retention priority, auditor, and reference labels. Increasing the cap reduces truncation from 74/367 (20.16%) to 31/367 (8.45%). M-F1 increases from 0.854 to 0.867 and U-F1 from 0.769 to 0.789; the reported M-to-U count decreases from three to two, while U-to-M remains one.

The baseline-truncated subset $T_{2048}$ contains 74 instances, including 13 reference M and eight reference U. Its M-to-U, U-to-M, and no-output counts are 1/1/1; Macro-F1 is 0.777, compared with 0.854 in the complementary 293 instances. This grouped difference is descriptive: cases requiring longer context may differ in difficulty. The whole-benchmark comparison does not establish which baseline-truncated instance accounts for the corrected M-to-U error, so no paired-subset causal effect is claimed.

One retained 367-instance independent-benchmark record contains 20 errors, including three no-output failures. Its run and seed cannot be identified from the retained records; the following error counts are not three-run means. Table S2a groups the mutually exclusive primary causes as applicability/routing (3), evidence/window (7), M/U boundary (4), and other (6). The last group includes implicit-measure interpretation, obligation granularity, and false V predictions. The independent benchmark does not measure the 27 unvisited reference obligations in the online path. Table S2b gives V counts by seed, and Table S2c summarizes FAIL-01 and FAIL-02 without adding them again to the aggregate counts.

#### 4.7.3. Runtime and Review Routing

The seed-42 ledger RUN-277E-3421O records 170 parsing calls and 277 processable events. Of these, 276 have ten final Pass clauses and one has nine, giving 2769 event-Pass-clause pairs. The 276 retrieved gold-standard applicable pairs expand to 340 obligations; the other 2493 system-Pass pairs expand to 3081. The online auditor, therefore, processes 3421 obligations, not 367. The 27 unvisited reference obligations remain failures in any complete online evaluation. Reference-obligation access is 340/367 = 92.64%; classification and UER for the full online output set are not supplied.

In this run, 276 of the 2769 system-Pass pairs matched the gold-standard applicable set (9.97%). System Pass denotes admission under the implemented exclusion checks rather than independently confirmed applicability; retained non-reference candidates increase the downstream audit workload. Table 8 summarizes the invocation counts and runtime calculation.

Stage means were measured in separate profiling trials with preloaded models, ten warm-up trials and 50 timed trials per stage, GPU synchronization, and batch size one. These profiles do not directly measure the online CE schedule with batch size 16.

Each of BM25, dense encoding/index lookup, and RRF processes 277 events; CE and the pre-guardrail process 11,080 pairs. CE uses batches of 16 separately for each 40-pair event pool, giving 277×⌈40/16⌉=831 forward passes; dense query encoding and neural auditing use batch size one. The serial cost calculation assumes preloaded models and excludes indexing, repairs, regeneration retries, and human review. The cost calculation reuses the profiling means as inputs. Multiplying them by actual call counts gives 29,299.3885 s. Adding an assumed 1% scheduling/output overhead yields an estimated 29,592.38 s (493.21 min; 8.22 h), or 174.07 s per review unit. Neural auditing accounts for most of this calculated cost. The estimate is distinct from direct wall-clock timing.

On the independent benchmark, 52/367 obligations require review (14.17%). These correspond to 38 tasks across 31/170 review units: 14 tasks contain two obligations and 24 contain one; seven units contain two tasks and 24 contain one. The 60 trigger occurrences comprise high risk (18), external materials (15), rule conflict (4), format-repair failure (1), untraceable evidence (7), candidate/evidence-rule conflict (12), and pre-routing/no-output review (3). Eight obligations have two triggers and none have three or more, giving 52 distinct flagged obligations. Automatic inputs route 62/367 obligations (16.89%). These are benchmark routing quantities, not online task counts or measured human-time savings.

## 5. Discussion

The pre-applicability guardrail controls admission of clauses to the audit evidence set. With verified inputs, Recall@10 increases from 0.898 to 0.928 and HCR@10 decreases from 0.312 to 0.041. Effective backfill accounts for 3.63% of final applicable hits. Table S1 also shows that limiting Unknown capacity can omit safety-related unresolved pairs, even when the Pass retrieval results are unchanged.

The post-evidence guardrail constrains determinate labels through label-specific support. Independent auditing yields UER 0.019 and FVR 0.016; V counts show fewer false positives after evidence checks, although two of six reference V cases remain undetected. With automatic inputs, independent-audit Macro-F1 decreases from 0.832 to 0.804. This deterioration and the 97 of 277 events requiring field correction indicate that extraction quality remains relevant to practical review.

### 5.1. Regulatory Interpretation and Traceable Review

Clause fragmentation is handled by retaining parent headings, version identifiers, and reference links in $H_{r}$; a fragment cannot establish an exception whose referenced premise is unavailable. Experts map normative terms to rule templates while preserving the responsible party, action, condition, and unit. Expressions such as “shall be ensured” may state an obligation without specifying an operational test; “reasonably practicable” requires contextual judgment; “as a general rule” invites exception analysis; and a “hazard condition” requires evidence that its trigger is met. “Is permitted” does not create an unconditional duty, and “the authority may” leaves discretion to the designated authority. Templates do not resolve these phrases by keyword matching alone. Missing premises or unresolved conflicts retain Unknown at applicability assessment or *U* at audit assessment.

FAIL-01 shows how unavailable evidence is mistaken for missing plan content. The plan states that the protection layout follows Figure A attached to the construction plan, which is absent from the input package. The clause ranks seventh after CE and fifth among final Pass results. All 1740 evidence tokens are retained, excluding truncation as the cause in this case. The reference is Pass/*U*, but both neural and post-guardrail outputs are *M* with no review flag. The unmarked attachment dependency defeats both stages; the appropriate action is *U* with a request for the attachment. In FAIL-02, verified event fields specify the preparation stage. A sixth-ranked clause applies through a cross-referenced exception, with reference audit label *S*. Missing exception metadata causes the pre-guardrail to Reject on the general stage condition; the auditor is not called and emits no S/M/V/U label. Increasing retrieval depth cannot repair this encoding error. Table S2 summarizes both cases. The stated corrective actions are required handling, not claims of completed review or repair.

The review record retains source and clause identifiers, source locations, applicable version, rule triggers, neural candidate, guardrail-adjusted label, review flag, and the reviewer’s decision and reason. Traceability supports checking hallucinated conditions and responsibility assignments but does not establish correct legal interpretation by itself. A regulatory update requires expert re-verification of affected conditions, exceptions, references, and obligations before a new repository-and-rule snapshot is frozen. The present experiments evaluate neither continuous regulatory updating nor universal legal reasoning. Final technical and approval responsibility remains with the authorized reviewers.

### 5.2. Limitations and Scope of Application

The evidence comprises five projects under one frozen regulatory regime. It supports within-regime project stability, not transfer across management systems or regulatory versions. Only six fixed-test references are V, so class-specific results remain descriptive. Field evaluation uses aligned events on presegmented review units and does not measure raw-document segmentation or event-discovery errors. The online run leaves 27/367 reference obligations unvisited; its complete classification score and UER are not established by the independent-audit benchmark. The runtime estimate includes an assumed 1% overhead, rather than a directly timed complete run. Benchmark routing of 52/367 does not measure human-time savings.

The method is limited to documentary review supported by available text. Drawings, field measurements, video, sensor records, and approval materials require separate evidence acquisition and validation. Work on large vision-language models (LVLMs) and LLM–BIM compliance checking [30,31], and LoFT-CLIP for few-shot railway-fastener anomaly detection [32], suggests possible future visual-evidence sources. Visual anomaly detection is a different task and supplies no validation of the present text-review metrics. Any extension must establish provenance and map visual findings to specific applicable obligations before they can support review.

## 6. Conclusions

This study addresses regulatory review of construction plans adjacent to operating railway lines by organizing the review process into three consecutive stages: candidate-clause discovery, clause applicability determination, and atomic-obligation fulfillment determination. On the basis of semantic candidate ranking, the pre-applicability guardrail forms an independent applicability determination using regulatory validity, construction discipline, construction stage, responsible party, spatial scope, and exception conditions. For Pass clauses that enter the review evidence set, the neural auditor produces candidate S/M/V/U states, and the post-evidence guardrail constrains determinate outputs using clause evidence and plan evidence. The method supports review routing for unresolved cases, with applicability-Unknown retention subject to the evaluated queue-capacity limit.

With verified event fields, fixed-test retrieval achieved Recall@10 0.928 and nDCG@10 0.878; independent auditing of 367 obligations achieved Macro-F1 0.832, UER 0.019, and FVR 0.016. The complete pre-applicability guardrail reduced HCR@10 from 0.312 to 0.041 with FRR 0.011. Automatic-input independent-audit Macro-F1 is 0.804. Field evaluation on 277 events gives measure-set micro-F1 0.899 and evidence-span F1 0.915, with 97 events requiring correction; N=25 retrieval yields Recall@10 0.914. The online ledger processes 3421 candidate obligations but accesses only 340/367 reference obligations. Complete online classification results remain unreported and cannot be replaced by independent-benchmark scores.

Under the same frozen regulatory repository and rules, the five project means yield Recall@10 0.926±0.008, Macro-F1 0.827±0.010, HCR@10 0.042±0.006, and UER 0.020±0.003. These results describe cross-project stability within that regime; they establish neither transfer to unseen regulations nor actual on-site fulfillment. The method supports traceable clause localization, documentary evidence checks, and professional review. It does not replace approval decisions. 

## Figures and Tables

**Figure 1:**
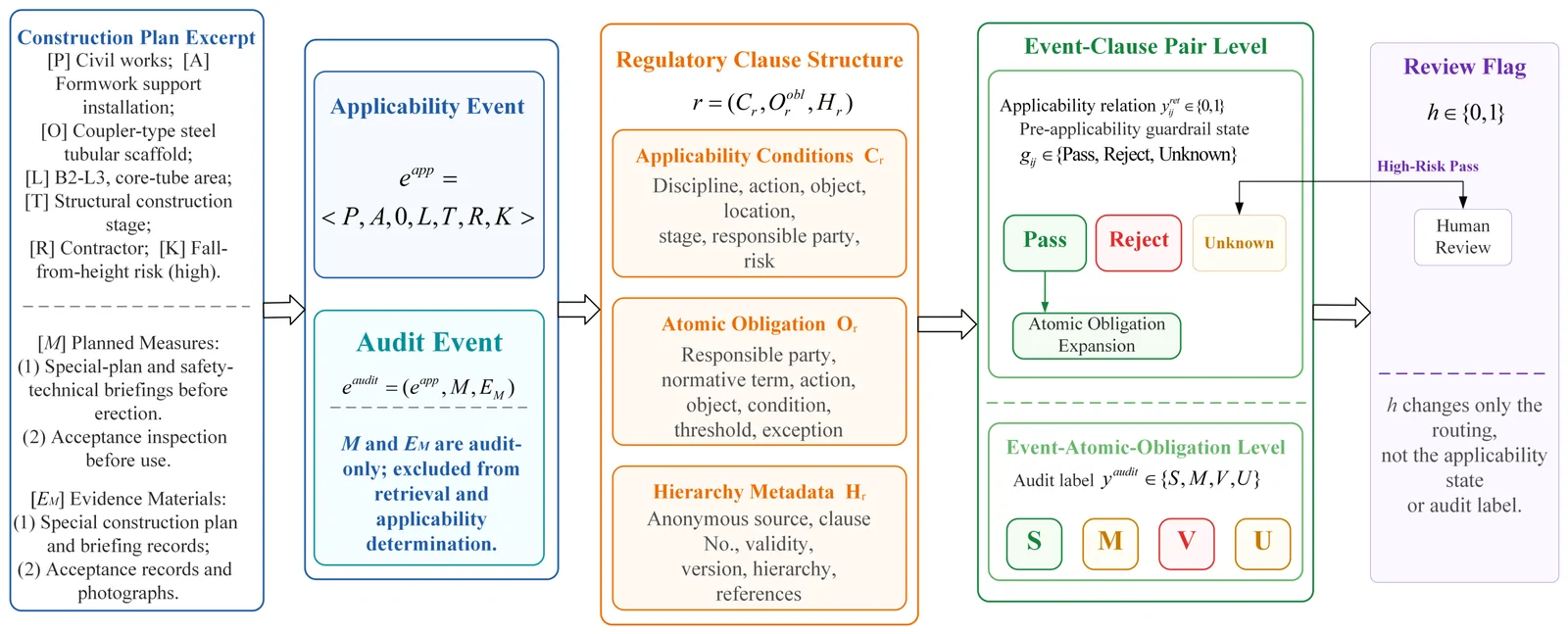
Construction events, regulatory clauses, and the labeling scheme. The plan excerpt is illustrative; measures and evidence locations are excluded from applicability inputs. Applicability states, obligation-level labels, and the review flag represent separate decisions. S, M, V, and U denote satisfied, missing, violation, and uncertain audit labels, respectively.

**Figure 2:**
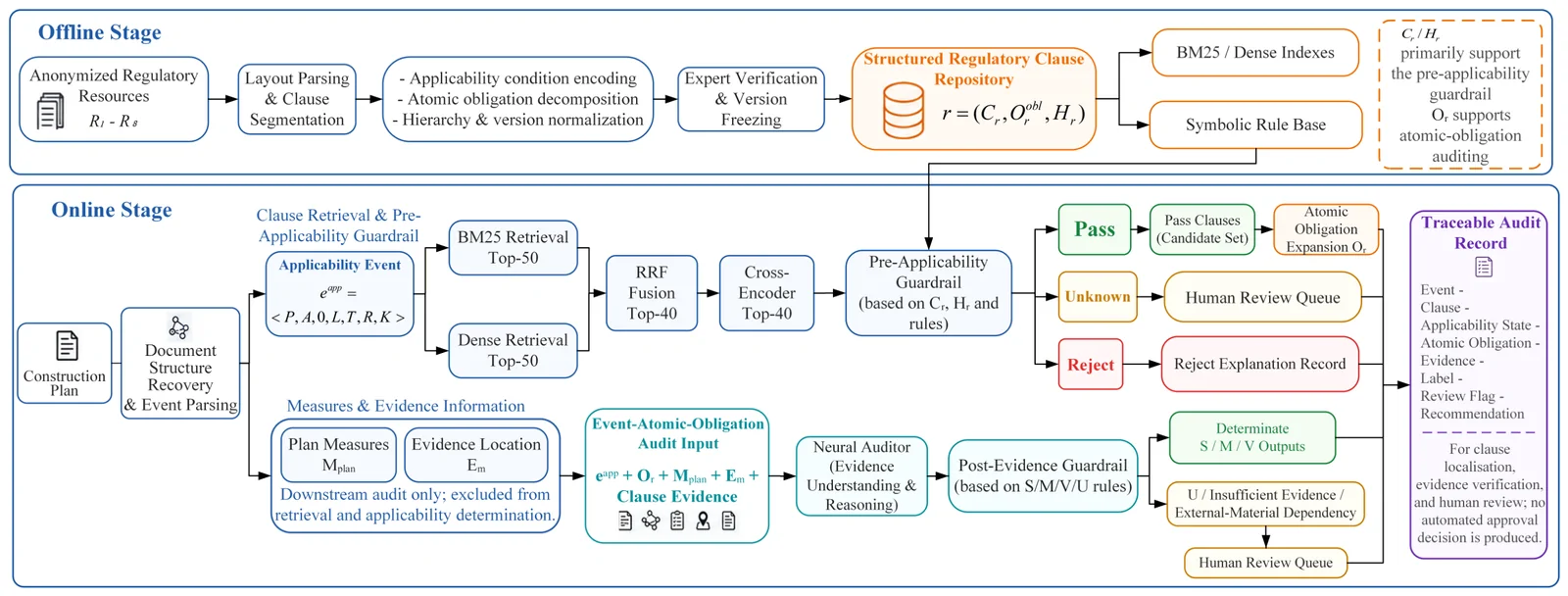
Overall framework of neuro-symbolic regulatory retrieval and compliance review. Offline knowledge preparation precedes online retrieval, applicability filtering, and obligation auditing. In this figure, $M_{\mathrm{plan}}$ and $E_{m}$ denote the measures set and its evidence locations. BM25: Best Matching 25; RRF: reciprocal rank fusion.

**Figure 3:**
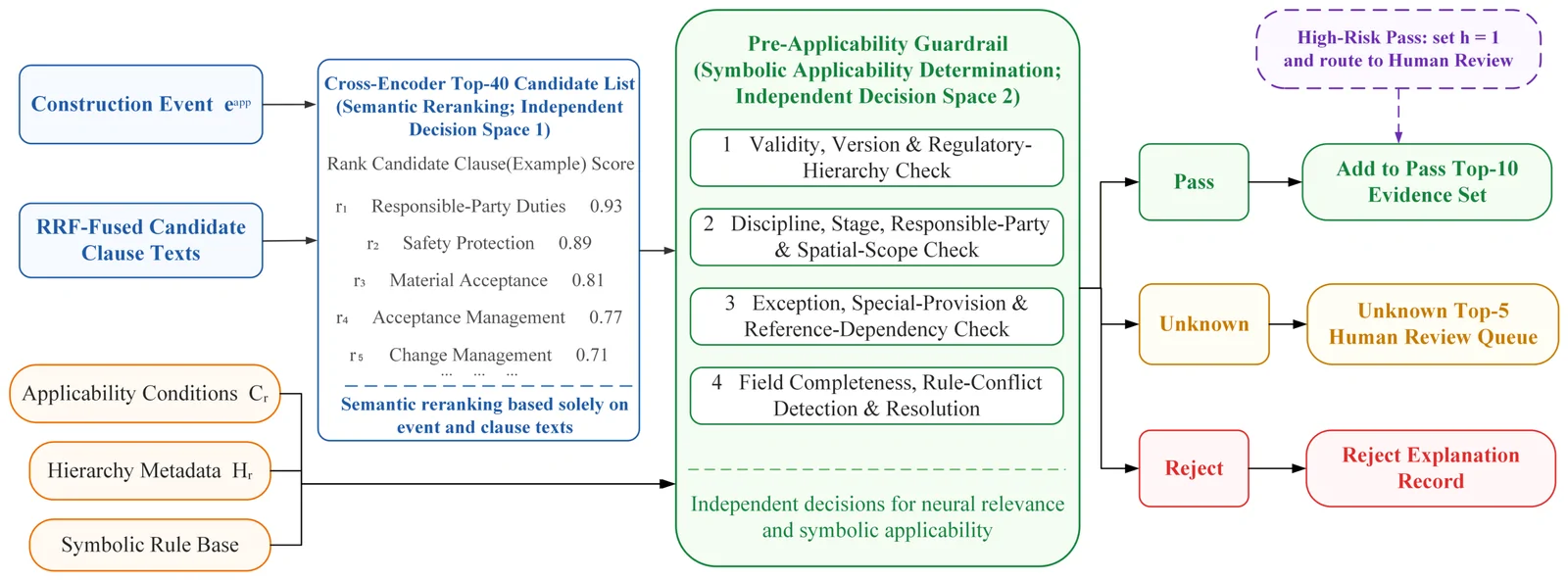
Neural reranking and the pre-applicability guardrail. Candidate descriptions and numerical scores are schematic examples, not experimental measurements. Pass candidates retain their ranking for Top-10 selection and backfill; Unknown Top-5 is a queue-capacity limit.

**Figure 4:**
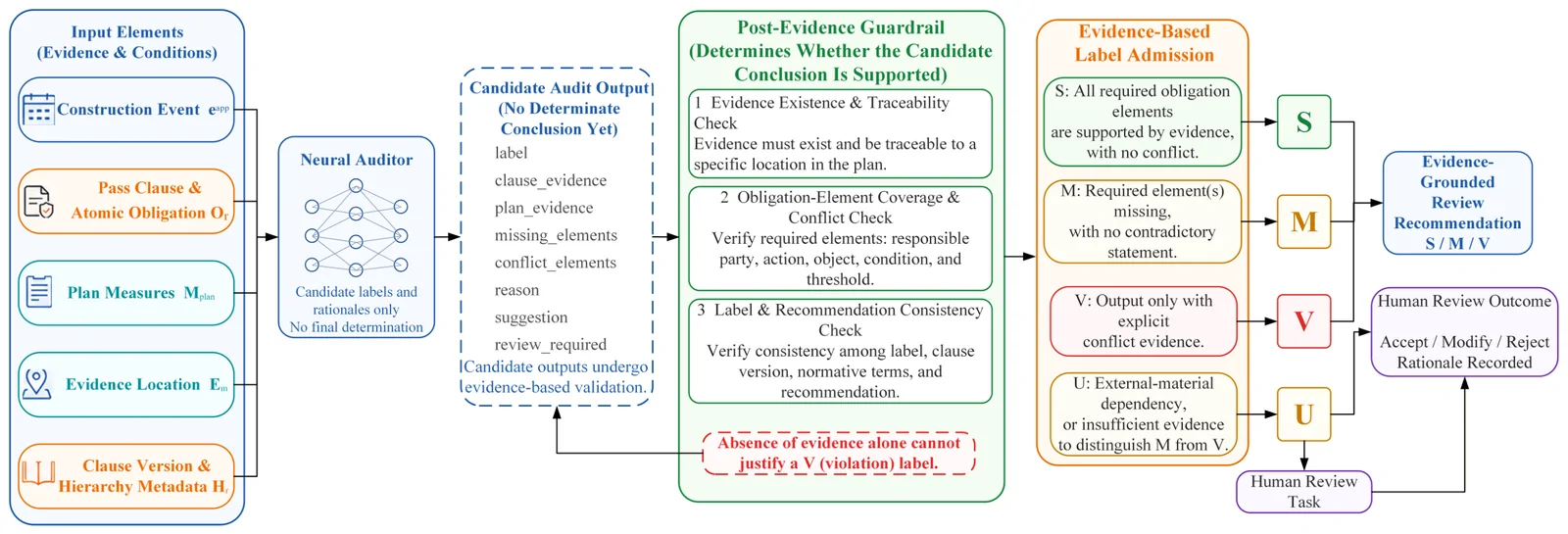
Neural auditing, the post-evidence guardrail, and human-review routing. For M, the evidence requirement concerns the examined scope and the identified missing element; an absent measure has no positive evidence span. The review flag remains separate from the audit label.

**Table 1:** Data scale of the five projects and the main fixed-split experiment.

Project	Fixed-Split Role	Documents	Review Units	Event-Atomic-Obligation Instances
Project 1	Training	6	155	355
Project 2	Validation	8	168	378
Project 3	Training	7	159	365
Project 4	Test	9	170	380
Project 5	Training	7	157	349
Total	–	37	809	1827

**Table 2:** Clause-retrieval results. Dense and Hybrid retrieve 243/297 and 256/297 applicable relations, respectively; their frozen retrieval outputs are identical across runs. BM25: Best Matching 25; nDCG: normalized discounted cumulative gain. ↓ indicates that lower values are better.

Method	Recall@10	nDCG@10	Mandatory-Clause Recall	Critical Miss Rate ↓
BM25	0.788	0.721	0.772	0.184
Dense	0.818	0.745	0.793	0.162
Hybrid	0.862	0.792	0.842	0.119
Cross-Encoder	0.898	0.840	0.881	0.087
Proposed	0.928	0.878	0.921	0.052

**Table 3:** Effects of the symbolic applicability guardrail. The three rule configurations incorrectly reject one, two, and three applicable pairs per run, respectively, each from 285 pool references. HCR: hard-conflict rate; FRR: false rejection rate; UCR: Unknown correctness rate. ↓ and ↑ indicate that lower and higher values are better, respectively.

Configuration	HCR@10 ↓	FRR ↓	UCR ↑	Recall@10
No guardrail	0.312	–	–	0.898
Validity rules only	0.238	0.004	0.800	0.903
Validity + discipline + stage	0.096	0.007	0.842	0.916
Complete pre-applicability guardrail	0.041	0.011	0.872	0.928

**Table 4:** Independent four-class review and evidence-consistency results on 367 gold-standard Pass instances with verified inputs. Available values are means over three runs; V-class counts are reported by seed in Table S2. Pre-only UER is omitted because its run-level numerator and denominator records could not be uniquely recovered. Complete online evaluation and routing are considered separately in Section 4.7. LLM: large language model; RAG: retrieval-augmented generation; UER: unsupported determinate-judgment rate; FVR: false violation rate. S, M, V, and U denote satisfied, missing, violation, and uncertain labels. ↓ indicates that lower values are better.

Method	Macro-F1	S-F1	M-F1	V-F1	U-F1	UER ↓	FVR ↓
Direct-LLM	0.520	0.917	0.527	0.286	0.350	0.165	0.066
Hybrid-RAG	0.680	0.953	0.703	0.500	0.564	0.086	0.033
Neural-only	0.739	0.961	0.764	0.615	0.615	0.065	0.033
Neural + Applicability Guardrail	0.771	0.968	0.818	0.615	0.684	–	0.033
Neural + Evidence Guardrail	0.800	0.971	0.800	0.727	0.700	0.028	0.016
Proposed	0.832	0.978	0.854	0.727	0.769	0.019	0.016

**Table 5:** Project-level retrieval and independent-audit cross-validation results. ↓ indicates that lower values are better.

Test Project	Recall@10	Macro-F1	HCR@10 ↓	UER ↓
Project 1	0.921	0.821	0.045	0.022
Project 2	0.934	0.837	0.036	0.017
Project 3	0.915	0.812	0.050	0.024
Project 4	0.926	0.830	0.042	0.019
Project 5	0.932	0.834	0.038	0.018
Cross-project mean ± standard deviation	0.926 ± 0.008	0.827 ± 0.010	0.042 ± 0.006	0.020 ± 0.003

**Table 6:** Field extraction and independent downstream benchmarks. Panel (**a**) uses 277 aligned test events; count triples are true positives/false positives/false negatives. Panel (**b**) juxtaposes retrieval and independent obligation-audit scores; it is not a complete online-pipeline comparison. First-pass Schema validity uses a separate 170-call denominator.

**(a) Field Outputs on the Fixed Test**						
**Field**	**Metric**	**Value**		**Count Basis**		
*P*: discipline	Accuracy	0.978		271/277		
*A*: activity	Set micro-F1	0.948		393/16/27		
*O*: object	Set micro-F1	0.955		331/12/19		
*L*: location	Accuracy	0.924		256/277		
*T*: stage	Accuracy	0.949		263/277		
*R*: responsible party	Set micro-F1	0.935		331/17/29		
*K*: risk category	Set micro-F1	0.910		292/20/38		
M: measures	Set micro-F1	0.899		612/50/88		
$E_{M}$: evidence	Character-span overlap F1	0.915		277 events; 14 missing		
Schema conformance	First-pass valid units/170	167/170		98.24%		
**(b) Retrieval and Independent Obligation-Audit Evaluation**						
**Input**		**Recall@10**	**HCR@10**	**Macro-F1**	**UER**	**Routing Rate**
Verified		0.928	0.041	0.832	0.019	52/367
Automatic		0.903	0.055	0.804	0.028	62/367

**Table 7:** Evidence-cap comparison on 367 reference obligations. Class F1 values are reported means; truncation and confusion counts describe one 367-instance evaluation population, not pooled three-run counts. The retained records do not identify the run or seed for these counts.

Evidence Cap	Truncated n/367	M-F1	U-F1	M→U	U→M
2048 tokens	74/367	0.854	0.769	3	1
3072 tokens	31/367	0.867	0.789	2	1

**Table 8:** Seed-42 online invocation counts and cost calculation. Each stage mean comes from 50 timed profiling trials after ten warm-up trials. Profiling and online batch settings are distinguished in the text. The final row is an estimate including an assumed 1% overhead.

Stage	Timing Unit	Actual Calls	Mean (s)
Event parsing	One parsing call	170	6.8000
BM25 + dense + RRF	One event query	277	0.0605
Cross-Encoder	One candidate pool	277	0.2400
Pre-applicability guardrail	One candidate pool	277	0.0040
Neural audit	One processed obligation	3421	8.2000
Post-evidence guardrail	One audit output	3421	0.0020
Complete pipeline (estimated)	One review unit	170	174.07

## Data Availability

The data presented in this study are not publicly available because they are derived from confidential railway-regulatory and project documents. All reported results are based on de-identified aggregate statistics. Access to the underlying documents and derived datasets is restricted by the data owner and cannot be publicly shared.

## Supplementary Materials

The following supporting information can be downloaded at: https://www.mdpi.com/article/10.3390/s26196076/s1, Table S1: Targeted retrieval and queue sensitivity. Panel (**a**) changes one parameter at a time around the main setting; *N* is each channel’s candidate count, *C* the fused pool size, and $k_{0}$ the RRF constant. Panel (**b**) uses Pass prefixes from the main candidate pool. Panel (**c**) distinguishes the 580 independent safety-unresolved references within CE Top-40 from all 600 in the complete reference universe; Table S2: Independent-audit errors and failure examples. Panel (**a**) groups 20 benchmark errors by mutually exclusive primary cause. Panel (**b**) reports V counts by seed on 367 reference obligations. Panel (**c**) summarizes two anonymized failures; required corrective handling is distinguished from an executed repair.

## Abbreviations

The following abbreviations are used in this manuscript:

AI	Artificial intelligence
NLP	Natural language processing
BIM	Building information modeling
IFC	Industry Foundation Classes
BM25	Best Matching 25
RRF	Reciprocal rank fusion
RAG	Retrieval-augmented generation
LLM	Large language model
LVLM	Large vision-language model
JSON	JavaScript Object Notation
FAISS	Facebook AI Similarity Search
NF4	4-bit NormalFloat
FP16	16-bit floating point
CPU	Central processing unit
GPU	Graphics processing unit
nDCG	Normalized discounted cumulative gain
HCR	Hard-conflict rate
FRR	False rejection rate
UCR	Unknown correctness rate
UER	Unsupported determinate-judgment rate
FVR	False violation rate
