# Supplementary material for: Neuro-Symbolic Regulatory Retrieval and Compliance Review for Railway Construction Plans Adjacent to Operating Lines
Source: Sensors (Basel). 2026 Sep 25;26(19):6076. doi: 10.3390/s26196076 (PMC13667735; doi:10.3390/s26196076)
Supplement: Supplementary file 1 [file sensors-26-06076-s001.zip › sensors-4531748-supplementary.pdf]

Supplementary Materials: Neuro-Symbolic Regulatory Retrieval and Compliance Review for Railway Construction Plans Adjacent to Operating Lines

Pengfei Guo, Yalong Xie, Zhihua Wang, Peng Zhi and Lamei Hu

Table S1. Targeted retrieval and queue sensitivity. Panel (a) changes one parameter at a time around the main setting; N is each channel's candidate count, C the fused pool size, and k_0 the RRF constant. Panel (b) uses Pass prefixes from the main candidate pool. Panel (c) distinguishes the 580 independent safety-unresolved references within CE Top-40 from all 600 in the complete reference universe.

(a) Candidate and RRF settings

Setting	N	C	k_0	Recall@10	HCR@10	Critical miss
Main	50	40	60	0.928	0.041	0.052
Fewer per channel	25	40	60	0.914	0.046	0.064
More per channel	100	40	60	0.929	0.043	0.051
Smaller fused pool	50	20	60	0.906	0.041	0.072
Larger fused pool	50	60	60	0.932	0.041	0.051
Smaller RRF constant	50	40	30	0.926	0.043	0.054
Larger RRF constant	50	40	90	0.928	0.041	0.052

(b) Rank-preserving Pass prefix

Prefix K	Recall@K	HCR@K	Critical miss@K
5	0.887	0.026	0.081
10	0.928	0.041	0.052
15	0.941	0.055	0.043

(c) Unknown queue capacity

Cap	Mean kept pairs/event	Retained reference pairs / 580 in Top-40	Reference pairs omitted by cap	Retained reference pairs / all 600
5	569/277=2.054	512/580=88.28%	45	512/600=85.33%
10	612/277=2.209	540/580=93.10%	17	540/600=90.00%
All	639/277=2.307	557/580=96.03%	0	557/600=92.83%

For $N = 25$, seeds 42/2024/2026 have 271/272/271 hits out of 297, 13/13/12 hard-conflict events out of 277, and 18/18/17 critical-miss events out of 277. Pooling gives 0.914/0.046/0.064, and mean nDCG@10 is 0.864. The smaller fused pool uses 20 actual candidates and the larger pool 60; the other listed configurations use 40. For the fused pool of 60, 42 critical-miss event records across three runs give 42/831=0.051. The main setting is retained for all subsequent comparisons.

In panel (c), 600 references comprise 557 correctly predicted Unknown, 20 outside Top-40, 17 misassigned Pass, and six misassigned Reject. The within-pool denominator is therefore 580. Capacity omissions are $557 - 512 = 45$, $557 - 540 = 17$, and $557 - 557 = 0$, respectively; the 43 upstream losses persist without a cap. Queue correctness uses all retained system pairs: 512/569=0.900, 540/612=0.882, and 557/639=0.872. The corresponding within-predicted-Unknown retention ratios, 512/557, 540/557, and 557/557, are not independent-reference coverage. The optional cap-20 source point gives 552/600=92.00% and does not replace the planned all-candidates comparison. The analysis measures retained pairs and does not assume that omitted records are subsequently recovered.

Table S2. Independent-audit errors and failure examples. Panel (a) groups 20 benchmark errors by mutually exclusive primary cause. Panel (b) reports V counts by seed on 367 reference obligations. Panel (c) summarizes two anonymized failures; required corrective handling is distinguished from an executed repair.

(a) Primary error cause

Cause	Count	Share of all errors
Retrieval or final selection	0	0%
Applicability or reference resolution	3	15%
Evidence access or context window	7	35%
M/U decision boundary	4	20%
Other	6	30%
Total errors, including no-output failures	20	100%

(b) V-class counts on the independent benchmark

Configuration	Seed	True positives	False positives	False negatives
Neural+Evidence	42	4	1	2
Neural+Evidence	2024	4	1	2
Neural+Evidence	2026	4	1	2
Proposed	42	4	1	2
Proposed	2024	4	1	2
Proposed	2026	4	1	2

(c) Two supplied failure cases

Record	FAIL-01	FAIL-02
Failure type	External evidence dependency	Exception metadata omission
Reference and outputs	Pass/U; neural M; final M; review flag false	Pass/S; pre-guardrail Reject; neural not called; no audit output
Evidence and rank	Referenced Figure A absent; 1,740/1,740 tokens retained; CE rank 7, Pass rank 5	Verified preparation-stage event; cross-referenced exception missing in metadata; CE rank 6
Cause and required handling	Unmarked external dependency; retain U and request the attachment	Omitted exception causes stage-based rejection; restore exception and reference chain

Panel (a) describes one retained 367-instance record without an identifiable run or seed; its error counts are not three-run means. It combines three pre-routing/no-output cases as applicability, five truncation plus two unavailable-material cases as evidence/window, four M/U-boundary cases, and six other cases. The zero retrieval count applies only to this independent-benchmark primary-cause grouping, not the complete online path. The online run has 27 unvisited reference obligations and requires its own error accounting.

In panel (b), both configurations at every seed predict V01–V04 as V, V05 as S, and V06 as U. All six rows have V-F1 $= 8/(8+1+2) = 0.727$; the one false positive comes from reference M, giving FVR $= 1/61 = 0.016$. Neural-only and pre-only each have 4/3/2, including one S-to-V and two M-to-V errors; post-only/Full improve V-F1 by reducing false positives while leaving the six reference-V predictions unchanged. Agreement on the six reference V predictions does not establish identical false-positive instances or complete error sets.

FAIL-01 is an unavailable-attachment error without truncation. FAIL-02 is a clause-level false rejection with no audit output, not an uncertain audit label. The case demonstrates one event–clause false rejection. The examples illustrate failure mechanisms and are not counted again in panel (a).
